# Tunable high-performance microwave absorption for manganese dioxides by one-step Co doping modification

**DOI:** 10.1038/srep37400

**Published:** 2016-11-17

**Authors:** Guocheng Lv, Xuebing Xing, Limei Wu, Wei-Teh Jiang, Zhaohui Li, Libing Liao

**Affiliations:** 1 Beijing Key Laboratory of Materials Utilization of Nonmetallic Minerals and Solid Wastes, National Laboratory of Mineral Materials, School of Materials Science and Technology, China University of Geosciences, Beijing 100083, PR China; 2 Department of Earth Sciences, National Cheng Kung University, Tainan, 70101, Taiwan; 3 Geosciences Department, University of Wisconsin – Parkside, Kenosha, WI 53144, USA

## Abstract

The frequencies of microwave absorption can be affected by the permanent electric dipole moment which could be adjusted by modifying the crystal symmetry of the microwave absorbing materials. Herein, we corroborate this strategy experimentally and computationally to the microwave absorption of manganese dioxides. Nanosized Co-doped cryptomelane (Co-Cryp) was successfully synthesized by a one-step reaction. The introduction of Co(III) induced a change of crystal symmetry from tetragonal to monlclinic, which could lead to an increase of its permanent electric dipole moment. As a result, the frequencies of maximum microwave absorption were regulated in the range of 7.4 to 13.9 GHz with a broadened bandwidths. The results suggested that microwave absorption of manganese dioxides can be tailored with Co doping to expand their potential uses for abatement of various microwave pollutions.

Electromagnetic pollution (EMP) is one of the largest pollution after air, water, and noise pollutions that threaten human’s health^1^. Thus, great efforts have been made to alleviate the EMP problem. As such, microwave absorbing materials (MAMs) become one of the hottest topics in the field of materials science for their potential reduction of EMP. In the 1980 s, MAMs made of ferrite were in the center of research focus^2^. The ferrite MAMs were replaced by MAMs made of polycrystalline fibers in America in the 1990s^3^. In the 21st century, nanoscale MAMs turn into a leading research focus in the world^4^,^5^,^6^,^7^,^8^.

Up to now, most of the researches have focused on developing new MAMs and increasing their microwave absorption rate. For example, magnetic nano-composites, such as ZnO/Fe^9^, α-Fe_2_O_3_@CoFe_2_O_4_^10^, and Ni/Co^11^, were extensively used as MAMs. The multi-walled carbon nanotube coated with CdS nanocrystals was a promising functional material for high temperature microwave absorption^12^. In the syntheses of BaMnZnCo-W ferrite, the influence of Co(II) content on the properties of microwave absorption was observed. At a sample thickness of 2.5 mm, a reflection loss of −40 dB could be achieved at a frequency of 11.5 GHz^13^. For the core-shell MnFe_2_O_4_-TiO_2_ nano-composites, their microwave absorbing properties were higher than that of MnFe_2_O_4_ when the permittivity and permeability of the complex MnFe_2_O_4_ and MnFe_2_O-TiO_2_ composites were measured in the microwave frequency ranges of 2–10 GHz^14^. In the 2 to 18 GHz range, 3-D Fe_3_O_4_ nanocrystals and multi-walled carbon nanotubes could enhance microwave absorption with tunable strong-absorption wavebands^15^. As a highly effective microwave absorption material, the Fe_3_O_4_/multi-walled carbon nanotubes are effective fillers for electromagnetic shielding and attenuation^16^. High-efficiency electromagnetic interference shielding could be achieved at elevated temperature using chemically graphitized r-GOs^17^.

However, the microwave absorption frequencies of all the MAMs mentioned above were not adjustable. Fewer studies were focused on regulating microwave absorption frequencies of the MAMs. Materials with high strength and adjustable absorption frequencies and bandwidth should have attracted more attention. The frequency that has the highest response to microwave absorption is the inherent property of the materials, which could be manipulated by controlling the crystal structure and morphology of the materials^18^,^19^. Because the basic properties of materials could affect magnetic and dielectric loss when the materials absorb microwave^20^, it is worth noting that crystal structure and morphologies are important factors to microwave absorption. In the frequency range of 4–14 GHz more than 99% of EM wave energy was attenuated by a synthesized architecture of Fe_3_O_4_ nanorod arrays and graphene sheets^8^. Moreover, MnO_2_ nanorods prepared with a hydrothermal method reached a minimum reflection loss of 74.1% or −5.9 dB at 7.57 GHz^21^.

Manganese dioxides (MOs) have excellent microwave absorption properties. They are made of corner- or edge-sharing [MnO_6_] octahedra mostly forming tunnel-like or layered structures^22^,^23^. Cryptomelane (Cryp) is a type of MOs made of a 2 × 2 tunnel structure. Cryp is able to produce instantaneous polarization and strong activity centre under the action of microwave^24^,^25^, form reactive oxygen species, and have strong responses to microwaves^26^. Furthermore, due to the replacement of Mn(VI) by Mn(III) and Mn(II), charge balance is compensated by K^+^ residing in the channels^27^. When different transitional metal ions enter the Cryp structure to substitute for Mn(III), a change of structure, morphology, or other physical properties could occur. Detailed changes could be linked to the types, amounts, and locations of the doped ions^28^. The structure of Co(II) doped Cryp is subject to change depending upon the Co(II) content^29^. With the change of crystal structure, the morphology and frequencies for microwave absorption are expected to change accordingly. In this study, the change of microwave absorption properties after Cryp was doped with different amounts of Co(III) was characterized. A one-step method to synthesis the Co(III) doped Cryp (Co-Cryp) that is able to regulate the microwave absorption frequencies and bandwidth was developed.

## Results and discussion

### Characterization of Cryp/Co-Cryp structure

**Figure 1:**
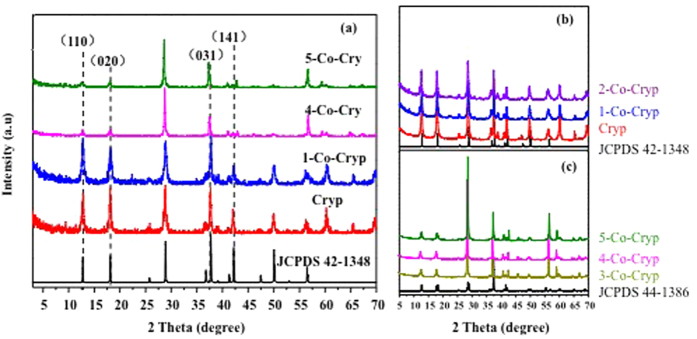
XRD of Co(III)-doped Cryp under different Co(III) concentrations (**a**) matching with monoclinic structure (**b**) and tetragonal structure (**c**).

The XRD pattern of raw Cryp matched well with the JCPDS 42–1348 (Joint Committee on Powder Diffraction Standards), indicating a tetragonal system with a space group of I4/m (Fig. 1a). The tetragonal structure is made of double chains of edge-sharing [MnO_6_] octahedra parallel to the c axis, and takes four such double chains with the same vertex angle forming a 2 × 2 tunnel framework^23^,^27^. As the amount of Co(III) doping increased, the XRD peaks of Co-Cryp became broader (Fig. 1b), and the structural symmetry changed to monoclinic with its XRD patterns matching well with JCPDS 44–1386 (Fig. 1c) when the amount of Co(III) doping was 3% or more.

**Figure 2:**
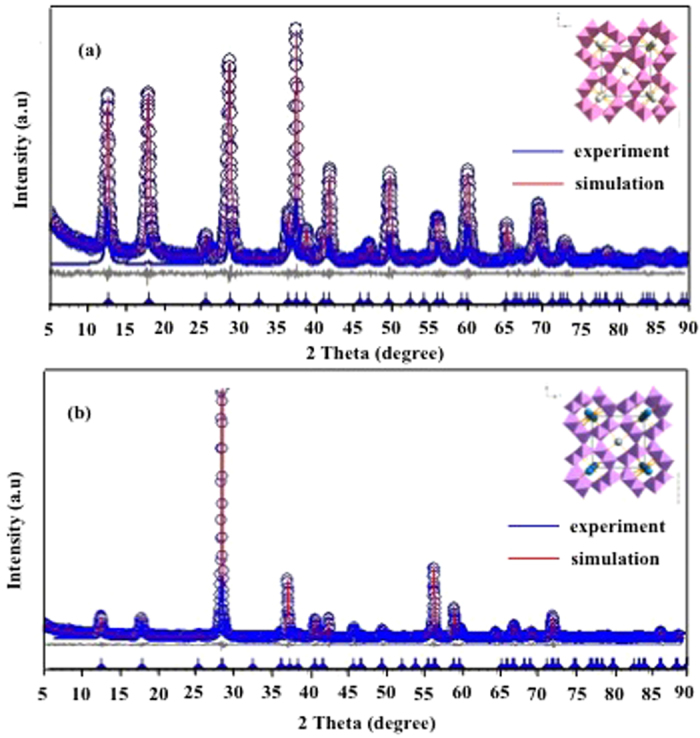
Structural refinement of Cryp with tetragonal symmetry and atomic coordinates (**a**) and monoclinic structure and atomic coordinates (**b**). 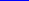 experiment, 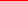 Rietveld simulation.

Occupancy of Co(III) the Mn sites was confirmed for both tetragonal and monoclinic structures of Co-Cryp in a structure refinement (Fig. 2). Modeling the atomic positions and site occupancies of the two Co-Cryp structures by the Rietveld method resulted in good discrepancy indices of R_exp_ = 8.14, R_wp_ = 11.35, R_p_ = 8.04, GOF = 1.39 (Table 1) and R_exp_ = 8.68, R_wp_ = 11.93, R_p_ = 7.59, GOF = 1.37 (Table 2), suggesting a tetragonal cell with 15% (atom) of Co and a monoclinic cell with 31% (atom) of Co, respectively. In fact, element analysis showed the molar ratios of Co(III) doped into Cryp. As the amount of Co(III) doping increased, more Co(III) was incorporated into the Co-Cryp. For 5-Co-Cryp and 4-Co-Cryp, The amounts of Co(III) were 2.5% and 2.1%, respectively (Table 3). As the amount of CoCl_3_ used in the synthesis of 5-Co-Cryp was two times that for 4-Co-Cryp, the minute difference in Co(III) contents between 5-Co-Cryp and 4-Co-Cryp suggested gradual saturation of Co(III) in 5-Co-Cryp.

**Figure 3:**
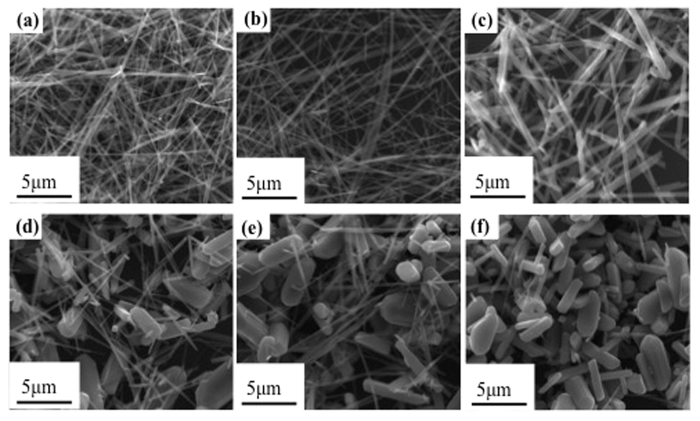
SEM of Cryp doped with different amounts of Co(III) (**a**): for raw Cryp; (**b–f**): for 1, 2, 3, 4 and 5-Co-Cryp.

**Figure 4:**
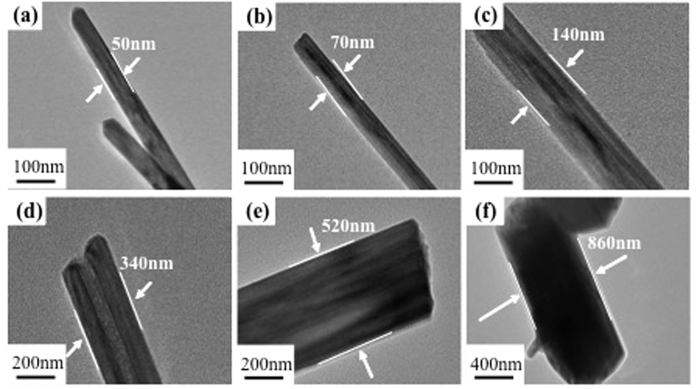
TEM of Cryp doped with different amounts of Co(III) (**a**): for raw Cryp; (**b–f**): for 1, 2, 3, 4 and 5-Co-Cryp.

The morphology of raw Cryp was nano-fibrous and the particle size was uniform, with a length of tens of micrometers and a diameter about 50 nm (Fig. 3a). As the doping amount of Co(III) increased, aggregates of granular particles micrometers in diameter became dominant (Fig. 3). The diameter of Co-Cryps changed from 50 nm to 860 nm as the amount of Co(III) increased (Fig. 4). In previous studies, the crystal morphology of Cryp changed from nanofibers to short rods or even granules and the degree of partial disorder of the Cryp structure increased^30^, as the doping of transition metal ions increased^31^,^32^.

**Figure 5:**
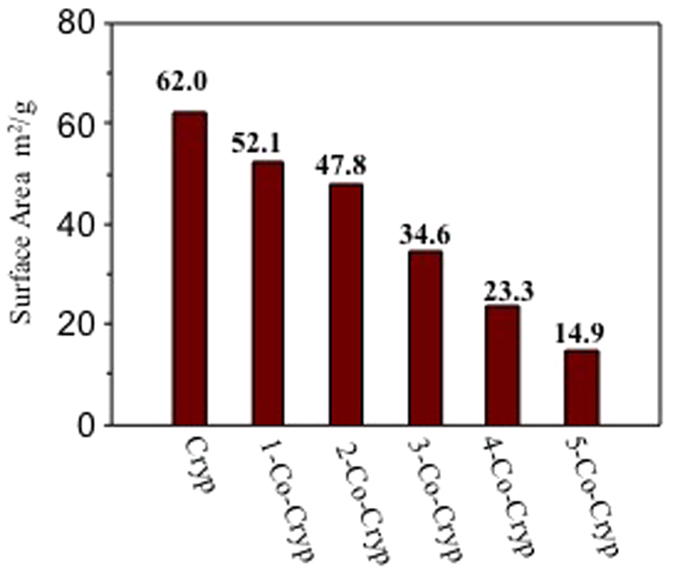
The SSA of Cryp with different amounts of Co(III) doping.

The specific surface area (SSA) of Cryp was determined using fully automatic nitrogen adsorption BET. As the amount of Co(III) doped increased, the SSA became smaller with the SSA of Cryp being 62.0 m^2^/g and that of 5-Co-Cryp being 14.9 m^2^/g (Fig. 5).

**Figure 6:**
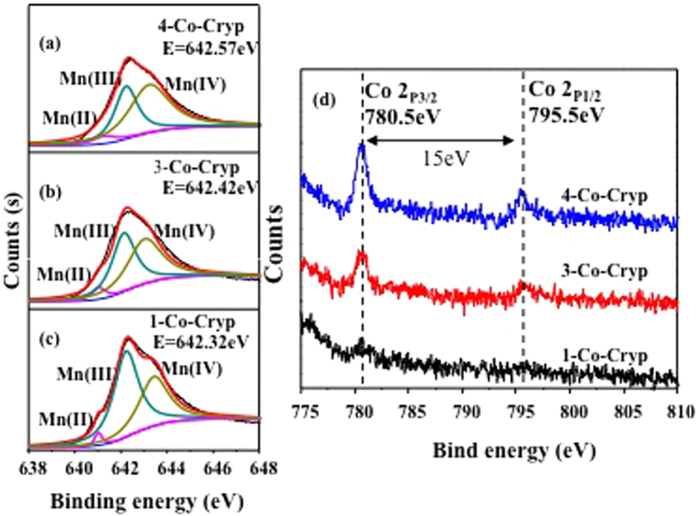
Mn (2p) and Co (2p) XPS spectra of Co-Cryp.

The Mn2p_3/2_ peak was deconvoluated by a multi-component spectral fitting to determine the distribution of various valence states of Mn in Co-Cryp (Fig. 6a–c). With the increase of initial Co input the amount of Mn(II) and Mn(IV) increased while that of Mn(III) decreased (Table 4). The Co(2p) XPS spectra of Co-Cryp showed that the binding energies of Co(2p_1/2_) and Co(2p_3/2_) were ca. 795.5 and 780.5 eV for all of the Co-Cryp samples (Fig. 6d). These values matched well with those of Co(III)^33^. In addition, the split of Co(2p_1/2_) and Co(2p_3/2_) was 15 eV which was nearly identical to that of Co(III) but 1 eV smaller than that of Co(II)^34^. On the contrary, Co(II) would show an observable satellite feature at 786 eV^35^, which is not visible in this study. These features pointed out the presence of Co(III), instead of Co(II), in the crystal structure of Co-Cryp. With Co(III) doping increased, the peak height of Mn(III) decreased accordingly, suggesting substitution of Co(III) for Mn(III) in Co-Cryp. In the preparation of Co-Cryp, Co(II) was oxidized to Co(III) due to the presence of excess Mn(VII) in the reaction. This resulted in substitution of Mn(III) by Co(III) in Co-Cryp, leading to relative increases of the Mn(II) and Mn(IV) signals (Fig. 6), particularly the latter, and thus a rise of the average oxidative state of Mn^36^.

### Microwave absorption properties of Cryp/Co-Cryp

Magnetic-dielectric composite materials were obtained by scattering Cryp and Co-Cryp particles into paraffin. Compared with metal composites, such Cryp- or Co-Cryp/paraffin composites had a higher resistivity. When a fixed amount of EM wave was applied on a material, a small amount of reflected EM wave would indicate an effective attenuation of EM wave energy and the material potentially has a good microwave absorbing property. A smaller coefficient (R) would indicate a larger absorption of the EM wave. The performance of the MAMs can be evaluated by a reflection R defined in the Supplementary Information section.

**Figure 7:**
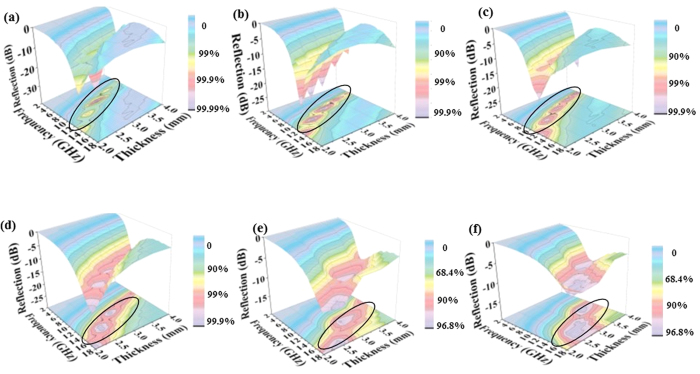
Microwave absorbing properties of Cryp and Co-Cryp. Microwave reflection losses of composites of paraffin and Cryp (**a**) and 1 (**b**), 2 (**c**), 3 (**d**), 4 (**e**), and 5-Co-Cryp (**f** ). Specimen thickness was in the range of 2 to 4 mm and the range of microwave frequency was 2–18 GHz.

The reflection losses of Cryp- and Co-Cryp/paraffin composites were calculated from equation 3 (SI) and Cryp/Co-Cryp accounted for 20% and paraffin for 80% of the total losses in Cryp- or Co-Cryp-paraffin composites (Fig. 7). Thus, the maximal attenuation of microwaves by the composites occurred as a function of both specimen thickness and microwave frequency (Fig. 7). With increasing specimen thickness, the maximal attenuation occurred at lower frequency (smaller *f*_*m*_ value) (SI: equation 3). In the thickness range considered, the width of frequency in which maximal microwave attenuation could be achieved by Co-Cryp was much greater than that by Cryp and it shifted towards higher frequency with increasing Co(III) doping. On the other hand, with increasing specimen thickness, the microwave absorption properties also increased.

**Figure 8:**
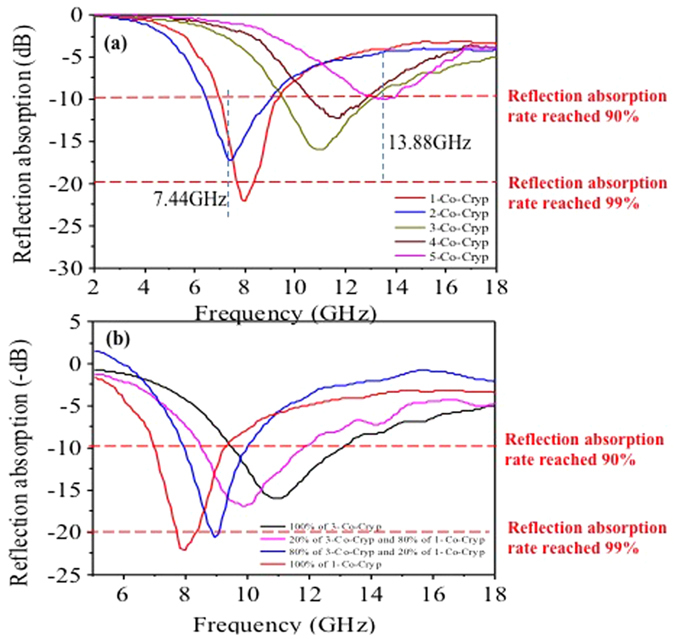
Microwave absorption properties of Cryp doped with different amounts of Co(III) (3 mm thick) (**a**); Microwave absorption properties of the mixture of 1 and 3-Co-Cryp (3 mm thick) (**b**).

Changes of EM absorption properties may stem from the phase transition of the MAMs^37^,^38^,^39^. The maximal reflection losses by the Co-Cryp composites mostly had less negative decibel values than −10 dB (i.e., 90% of the incoming power was absorbed), in comparison to −35.4 dB (>99% power absorbed) for the Cryp composite at 3 mm composite thickness (Fig. 7a). An increase of Co(III) doping resulted in a phase transition from tetragonal to monoclinic structure and a decrease (less negative decibel value) of reflection loss and energy absorption (Fig. 8a). However, the frequency and bandwidth for EM attenuation became larger as the Co(III) doping increased (Fig. 8b). This feature suggests potentially broader uses of the functional materials for EMP attenuation^40^.

**Figure 9:**
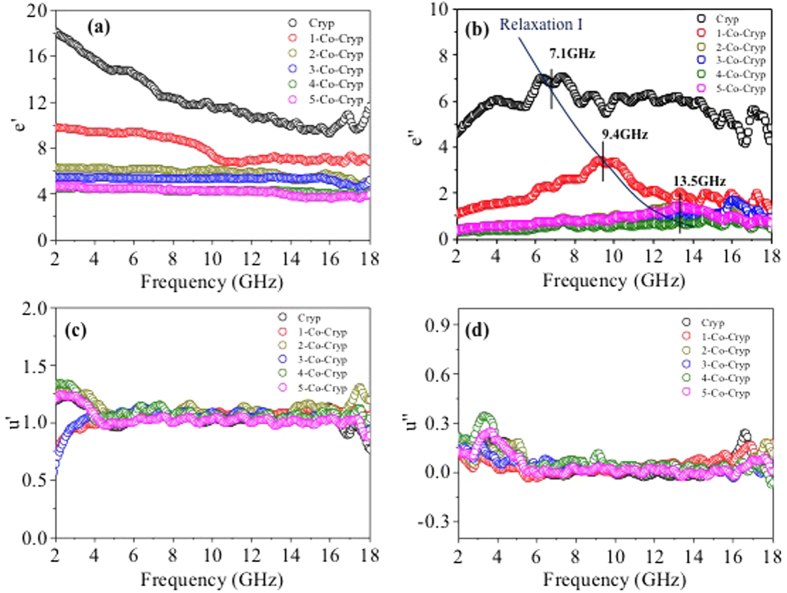
Relative complex permittivity of the real (**a**) and imaginary (**b**) parts and relative complex permeability of the real (**c**) and imaginary parts (**d**) of Cryp doped with different amounts of Co(III) in the frequency range of 2–18 GHz.

Microwave absorption was attributed to magnetic loss and dielectric loss^41^. Permittivity mainly originates from polarizations of electrons, ions, and intrinsic electric dipoles. Magnetic properties could be affected by crystal structure, special geometrical morphology and size^42^,^43^. Complex permittivity and complex magnetic permeability of Co-Cryp were analyzed (Fig. 9). The real (ε′) and imaginary (ε″) complex permittivity, and the real (μ′) and imaginary (μ″) permeability are displayed in Fig. 9a–d. As the amounts of Co(III)-doped increased, the ε′ became smaller, suggesting gradual weakening of the dipole polarization and electric polarization of Co-Cryp. Because ε′ represents material characterization of polarization. It could clearly be seen the values of ε″ exhibited very complex nonlinear behaviour^44^, and also decrease with the increase of Co(III) doping in Cryp. As shown in Fig. 7b, there were relaxations of Cryp. Relaxation I, was located at the frequencies of 7.1, 9.4, and 13.5 GHz for raw Cryp, 1-Co-Cryp, and 2-Co-Cryp, respectively. The relaxation of Cryp was mainly from interfacial polarization and defect dipole polarization^39^,^45^,^46^. As the Co(III) doping increased, the frequency of relaxation I shifted to higher frequencies. Compared with the complex permittivity, the changes in complex magnetic permeability were minute and close to zero. Thus, the complex permittivity was the key factor affecting microwave absorption of Cryp.

### The change of crystal symmetry and permanent electric dipole moment

The radii of Mn(III) and Co(III) are 0.66 and 0.63 Å. Due to the size difference, the introduction of Co(III) could induce some distortions in the [MnO_6_] octahedra, resulting in a decrease of crystal symmetry from tetragonal to monoclinic^47^,^48^ (Table 5). Accordingly, the anisotropicity increased^20^ and the morphology changed from nano-fibers to equidimentsional granules of micrometers in size^49^,^50^,^51^ (Figs 3 and 4). Nanosize materials tend to have better microwave absorption properties^52^,^53^,^54^,^55^. As evidenced in this work, the nanosize Cryp fibers showed greater EM absorption than the micrometer-sized Co-Cryp.

Moreover, the permanent electric dipole moment increased from 0 to 3.3792 × 10^−29^ C·m per [MnO_6_] octahedron with the increase of anisotropicity (Table 6). The permanent electric dipole moment is closely related to the composite magnetic permeability and complex dielectric constant of materials (Equations 2–4 in SI), and could cause different *f*_*m*_ values and absorption bandwidths^56^,^57^,^58^,^59^ as is the case in this study. With an increase in the amount of Co(III) doping, a right shift of *f*_*m*_ occurred and the bandwidth of maximal microwave attenuation became significantly larger, albeit with a slight decrease of absorption performance. These features make it optimistic to use Cryp or Co-Cryp as MAMs in a wide range of environments for potential microwave abatement^60^,^61^,^62^.

## Conclusions

Cryp was demonstrated to be a very good microwave absorption material. The micowave reflection loss of Cryp reached to −35.4 dB. As the amount of Co(III) doped increased, the bandwidth of high attenuation notably expanded and the frequencies shifted significantly to 13.9 GHz. In a nutshell, optimal microwave absorption frequency and bandwidth of Cryp materials can be tuned with a control of crystal structure via selective Co doping.

## Experimental Section

### Experimental materials

KMnO_4_, MnSO_4_•H_2_O, CoCl_2_•6H_2_O, diethyl ether, paraffin, and distilled water were purchased from Beijing Chemical Works. All chemicals were analytical grade.

**Synthesis of Cryp and Co-Cryp **

The Cryp samples were produced by the following procedures: 100 mL of 0.1 M KMnO_4_ and 50 mL of 0.3 M MnSO_4_•H_2_O were mixed at room temperature under vigorous stirring for 30 min. Gradually formed brwon precipitates were transferred into a stainless steel autoclave lined with Teflon, and were cured at 140 °C for 24 h. After been being cooled down to room temperature, the precipitates were filtered first, then, washed three times with distilled water and dried at 80 °C for 24 h^63^.

The Co-Cryp samples were produced similarly with different quantities of CoCl_2_•6H_2_O added to the MnSO_4_•H_2_O solution before being mixed with the KMnO_4_ solution. During the reaction, Co(II) was oxidized to Co(III) due to an excess amount of Mn(VII) with respect to the reduced amounts of Mn(II) in the reaction.

### Preparation of Cryp/Co-Cryp-paraffin composites

The composites for microwave absorption measurement were fabricated by mixing Cryp or Co-Cryp (20%) with paraffin matrix (80%). The mixtures were pressed into a cylindrical shape with *Φ*_*out*_ =7.00 mm and *Φ*_*in*_ =3.04 mm, respectively. The reflection losses of the composites should be attributed to that of the Cryp or Co-Cryp as the reflection loss of paraffin matrix was similar to that of the air.

### Methods of analyses

The powder X-ray diffraction (XRD) patterns of Cryp and Co-Cryp were recorded with a CuKα radiation at 40 kV and 100 mA, a scanning speed of 8° 2θ/min with 0.02° per step. To further investigate the changes in crystal structure of Cryp, Rietveld refinement was performed using the Topaz 3.0 program with the structural parameters of Cryp used as initial parameters.

The purities of the final products were carefully checked by XRD analysis from 5° to 90°. Step scan performed in structural analysis at a rate of 2.35 s/step and a step size of 0.02. TOPAS package was deployed to calculate the structural details in the Rietveld refinement.

Elemental composition of Cryp was determined by X-ray Fluorescence spectrometry (XRF). Micro textures of the Cryp and Co-Cryp precipitates were observed on an FEI Quanta 250 field-emission environmental scanning electron microscope (ESEM) with a voltage of 15 kV. Samples were prepared by drying thick sample suspension on a silicon slide before the ESEM observation. Detailed microstructures and crystallite sizes were analyzed by high-resolution transmission electron microscopy (Model: JEOL JEM-2010F).

An X-ray photoelectron spectroscopy (XPS, Thermo Scientific Co., Ltd.) was used to determine the surface valence of Cryp after being doped with different quantities of Co(III) under a monochromatic CuKα source at 150 W and a base pressure of 6.5 × 10^−10^ mbar in the measuring chamer.

The permittivity and permeability of the composites were measured by a coaxial wire method in the 2–18 GHz range using a phasor network analyzer PNA N5244A (Agilent).

The formula for the calculation of the geometric centre follows that for the calculation of the permanent electric dipole moment^64^.


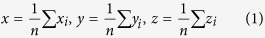



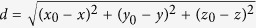
 is the deviation of coordinates of the central atom from the geometric centre. For the [MnO_6_] octahedron:


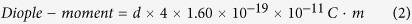


## Additional Information

**How to cite this article**: Lv, G. *et al.* Tunable high-performance microwave absorption for manganese dioxides by one-step Co doping modification. *Sci. Rep.* **6**, 37400; doi: 10.1038/srep37400 (2016).

**Publisher’s note:** Springer Nature remains neutral with regard to jurisdictional claims in published maps and institutional affiliations.

## Supplementary Material

Supplementary Information

## Figures and Tables

**Table 1:** Coordinates and occupancies of atoms in tetragonal Cryp.

R_exp_: 8.14		R_wp_: 11.35	R_p_: 8.04	GOF: 1.39	
R_exp_′: 4.14		R_wp_′: 5.78	R_p_′ : 4.37	DW: 1.01	
Atom	Position	Occupancy	x	y	z
K	4	0.33	0	0	0.4959
Co	8	0.15	0.3528	0.1698	0
Mn	8	0.85	0.3528	0.1698	0
O1	8	1	0.1589	0.1825	0
O2	8	1	0.5214	0.1699	0

**Table 2:** Coordinates and occupancies of atoms in monoclinic Co-Cryp.

R_exp_: 8.6		R_wp_: 11.93	R_p_: 7.59	GOF: 1.37	
R_exp_′: 6.30		R_wp_′: 8.65	R_p_′ : 5.66	DW: 1.1	
Atom	Position	Occupancy	x	y	z
K	4	0.33	0	0.3754	0
Co	8	0.31	0.2837	0	0.0993
Mn	8	0.69	0.2837	0	0.0993
O1	8	1	0.1750	0	0.2282
O2	8	1	0.7925	0	0.3072

**Table 3:** The elements of Co-Cryp by the method of XRF.

	Co	Mn	O	K
Cryp	0	73.3%	24.3%	2.4%
1-Co-Cryp	0.23%	72.6%	24.6%	2.5%
2-Co-Cryp	0.52%	72.0%	24.7%	2.7%
3-Co-Cryp	1.6%	70.9%	24.7%	2.7%
4-Co-Cryp	2.1%	70.1%	24.8%	2.7%
5-Co-Cryp	2.5%	69.3%	25.4%	2.6%

**Table 4:** Results of XPS analysis.

Catalyst	Manganese distribution		
	Mn(II)	Mn(III)	Mn(IV)
1-Co-Cryp	2.57%	58.23%	39.20%
3-Co-Cryp	5.24%	45.36%	49.40%
4-Co-Cryp	5.37%	36.50%	58.13%

**Table 5:** The cell parameters of Cryp.

Crystal system	a/Å	b/Å	c/Å	β
Cryp-T(I4/m)	9.8401	9.8401	2.8619	90
Cryp-M(C2/m)	9.8317	2.8678	9.8233	90.95

**Table 6:** The coordinates of atoms and permanent electric dipole moments of Cryp.

Cryp-T	Unit (nm)			Cryp-M	Unit (nm)		
	a	b	c		a	b	c
Mn	0.8489	0.6700	1.5	Mn	0.1646	0.5	0.34695
O1	0.8350	0.5421	2	O1	0.0423	0	0.3249
O2	0.8350	0.5421	1	O2	0.0423	1	0.3249
O3	0.8456	0.7971	1	O3	0.2947	0	0.3499
O4	0.8456	0.7971	2	O4	0.2947	0	0.3499
O5	0.6545	0.7030	1.5	O5	0.2053	0.5	0.1501
O6	1.0421	0.6650	1.5	O6	0.1561	0.5	0.542
PEDM	d_1_	About 0		PEDM	d_2_	0.528	
Dipole moment (d*q)		0		Dipole moment (d*q)		3.3792*10^−29^ C∙m	
