# Supplementary material for: Tunable high-performance microwave absorption for manganese dioxides by one-step Co doping modification
Source: Sci Rep. 2016 Nov 17;6:37400. doi: 10.1038/srep37400 (PMC5112597; doi:10.1038/srep37400)
Supplement: Supplementary Information [file srep37400-s1.doc]

Supplementary Information

Tunable high-performance microwave absorption for manganese dioxides by one-step Co doping modification

Guocheng Lv^1^,[[1]](#footnote-2), Xuebing Xing^1^, Limei Wu^1^, Wei-Teh Jiang^2^, Zhaohui Li^1,2,3,*^, Libing Liao^1^

The electromagnetic performance was tested using the coaxial method in N5244A (Agilent). The measuring principle was based on the transmission/reflection model proposed by Nicolson^1^. While the thickness of sample was infinite, Reflection coefficient of samples that have absorbed the electromagnetic wave was calculated by:


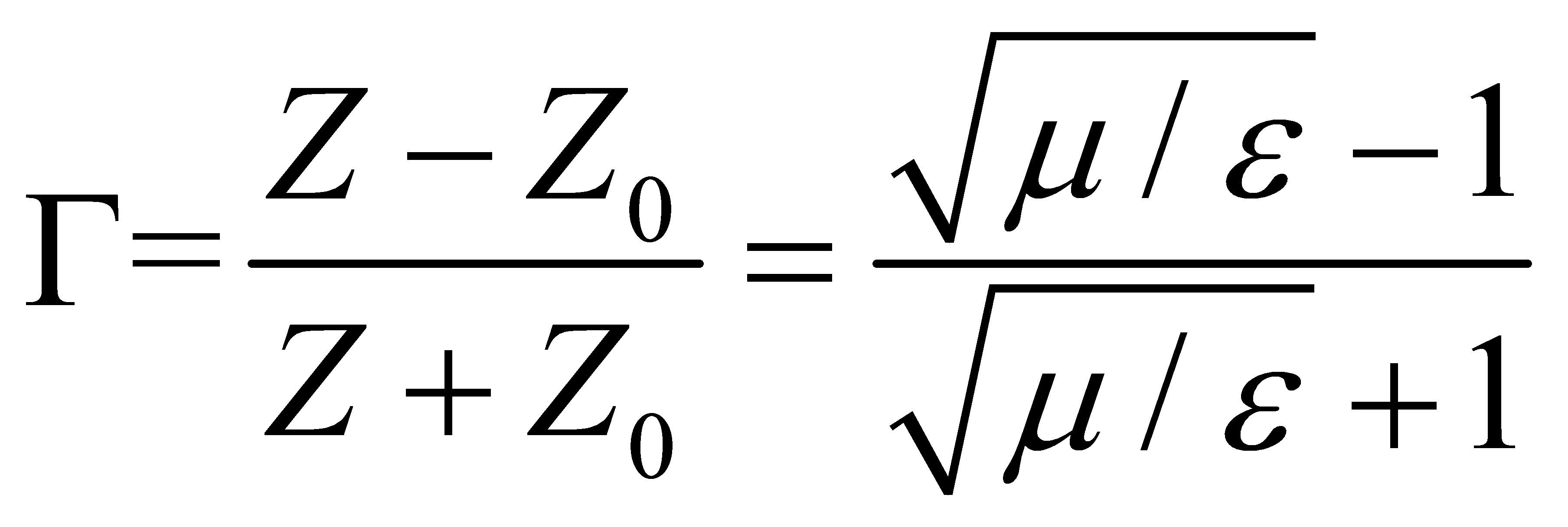
 (1)

where *Z_0_* is impedance of the air line, *Z* is impedance of sample, *μ* and *ε* were the composite magnetic permeability and complex dielectric constant, respectively. With a thickness of sample L, the transmission coefficient of the sample is calculated by:


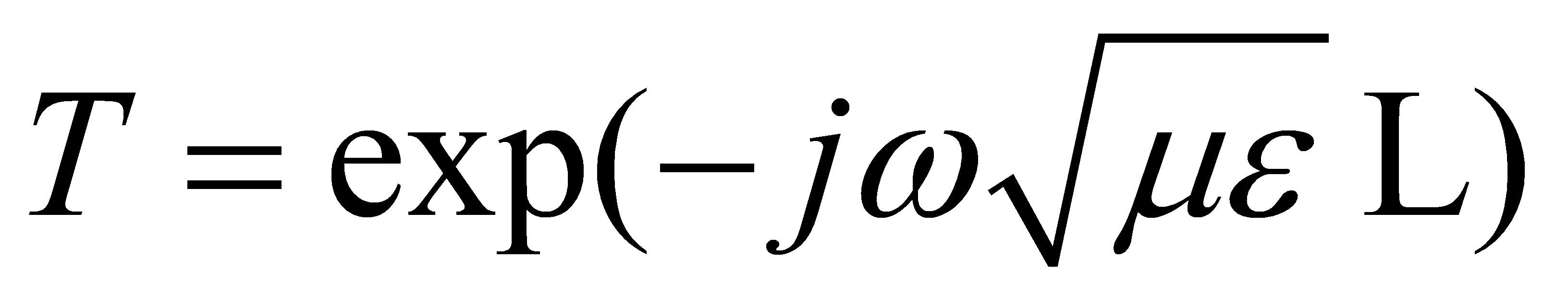
 (2)

According to equation (1) and (2), composite magnetic permeability and complex dielectric constant of materials were determined by:


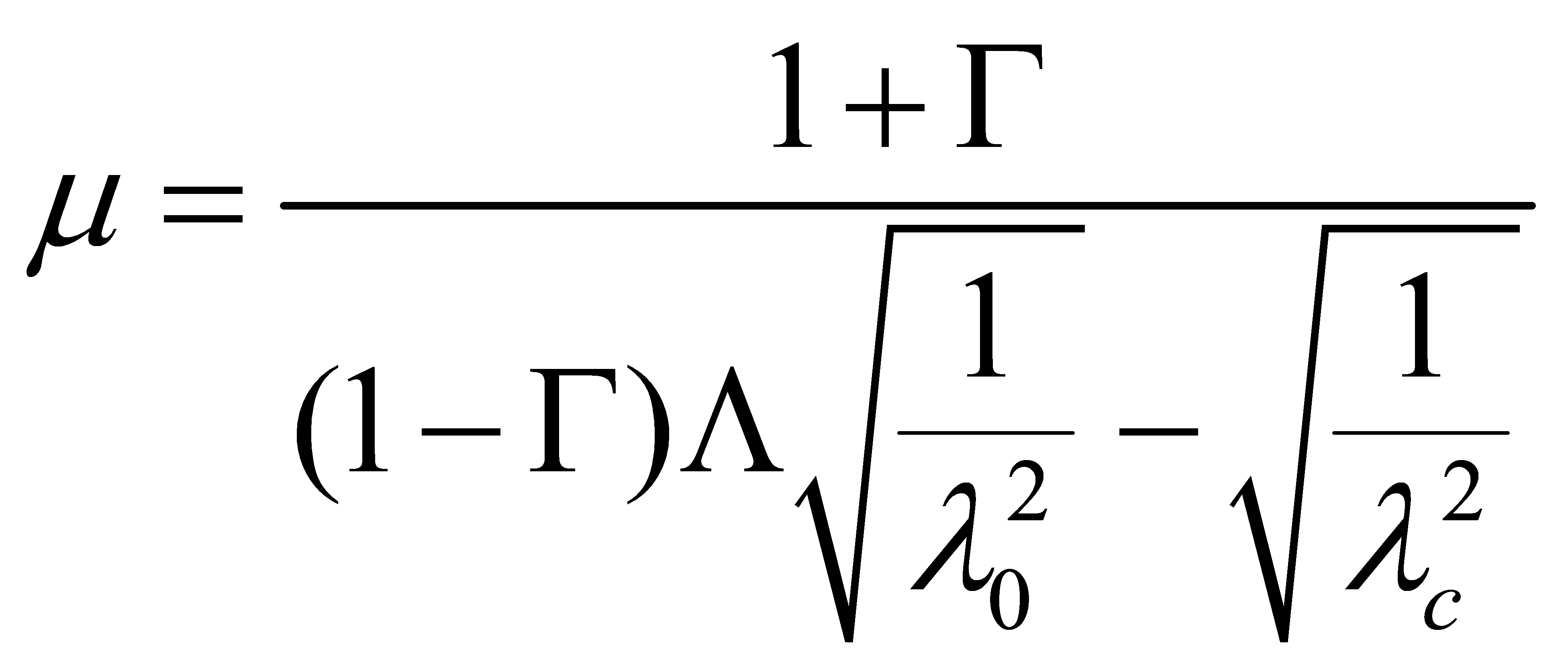
 (3)


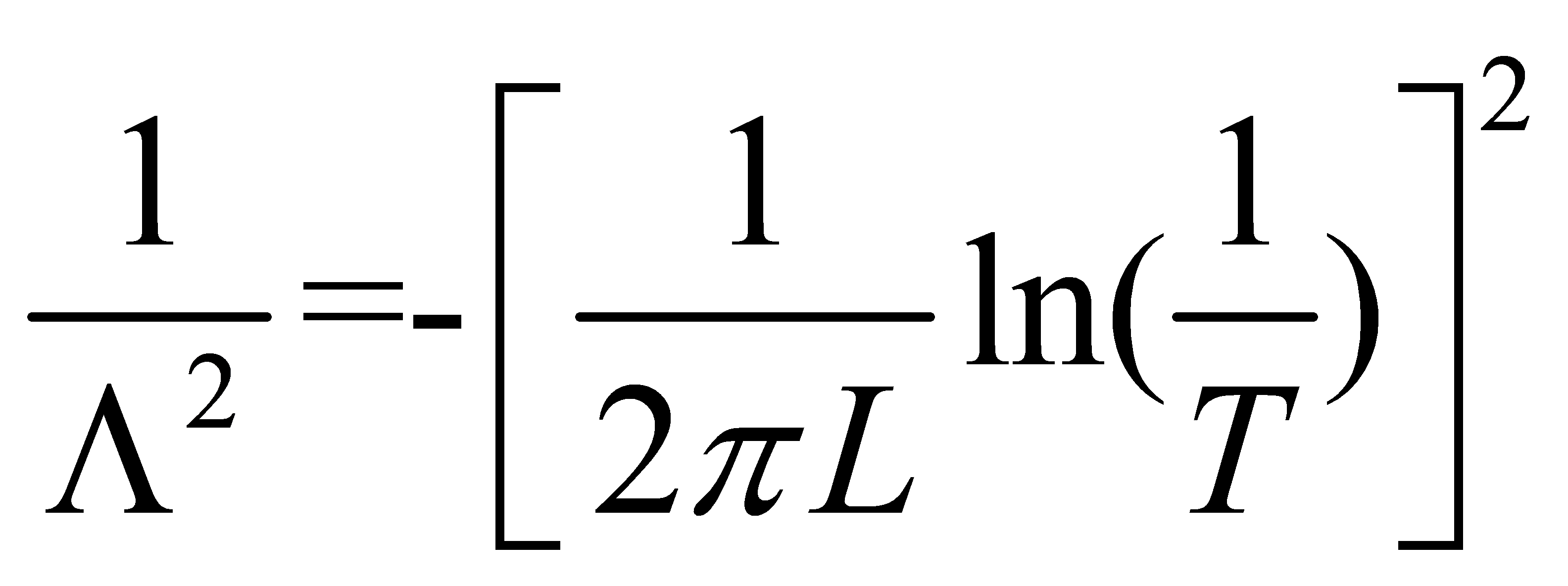
 (4)


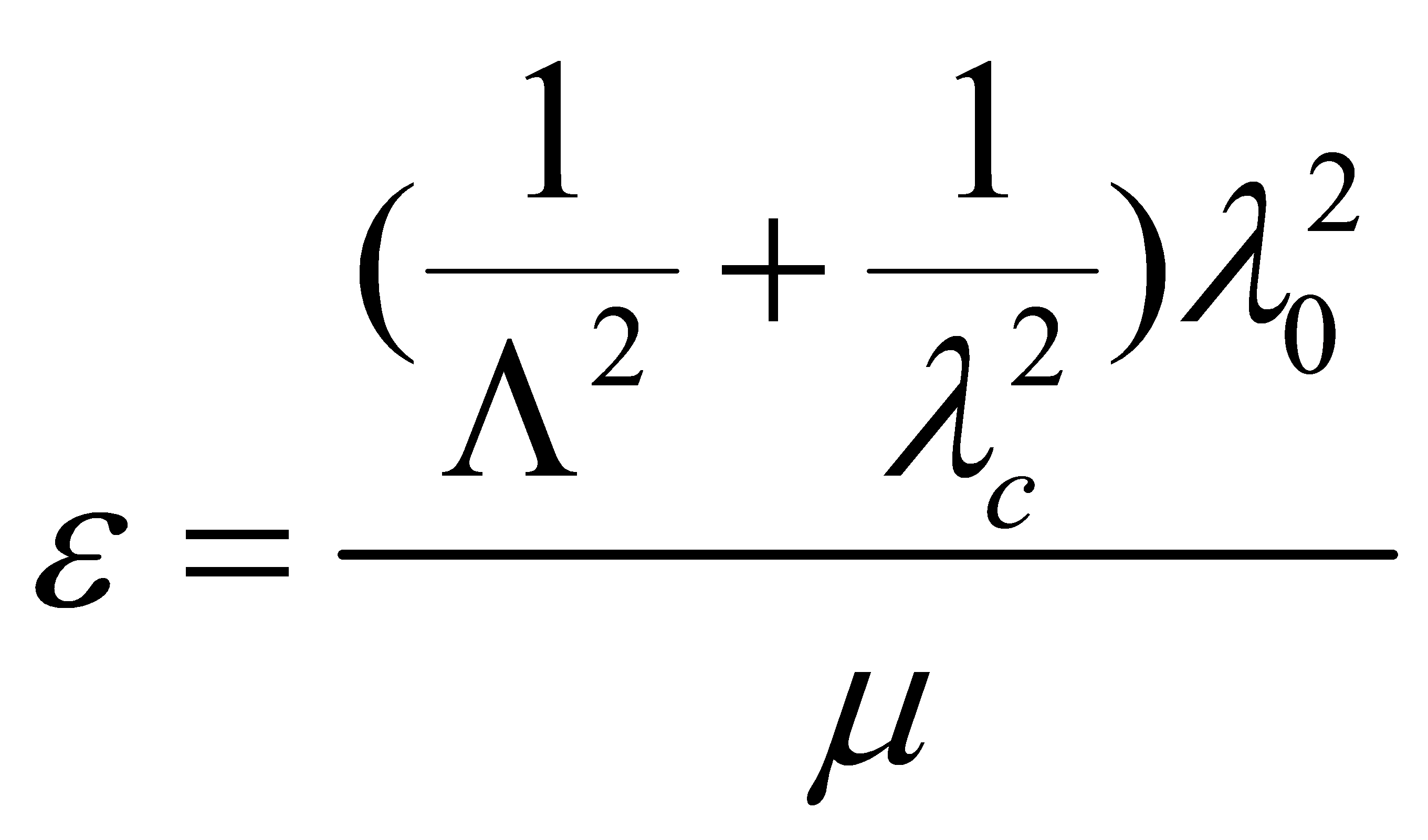
 (5)

The performance of the MAMs can be judged by a reflection coefficient (R) defined as follows^2^:

(6)

(7)

(8)

where *Z_0_* = 377 Ω, is the impedance of the air, *Z_j_* is input impedance, *μ_0_* and *ε_0_* are magnetic permeability and dielectric constant in vacuum, and *μ* and *ε* are those of the composites in air, *f* is the frequency of an EM wave, and *d* is the composite thickness. Thus, a smaller *R* would indicate a larger absorption of the EM wave.

When EM wave was irradiated on the surface of materials, the matching frequency, *f_m_* which was the frequency of minimal reflection coefficient (maximal attenuation) having a relation with the specimen thickness, *d* by:

(9)

where *c* is the speed of light.

The Co-Cryp was the tetragonal or monoclinic depending on the amount of Co(III) doped. 1-Co-Cryp, 3-Co-Cryp, and 4-Co-Cryp were analyzed by XPS. Electron binding energy of Mn2p_3/2_ in was 642.32, 642.42, and 642.57 eV for 1-Co-Cryp, 3-Co-Cryp, and 4-Co-Cryp, respectively. As higher electron binding energy indicated higher oxidizability of Mn and lower electron binding energy indicated lower oxidizability^3^, it was deduced that the [average](http://dict.cnki.net/dict_result.aspx?searchword=平均&tjType=sentence&style=&t=average) oxidizability of Mn in Co-Cryp could be increased as the amount of Co(III) doped increased, which suggested more Mn(IV) in 4-Co-Cryp. In the preparation of Cryp, Co(II) was oxidized to Co(III) by the redundant Mn(VII). This may suggest that Mn(III) was replaced by the Co(III) in Co-Cryp, leading to a relative increase in Mn(II) and Mn(IV), thus, an increase in the average oxidizability of Mn^4^.

References:

# 1. Nicolson, A. M., & Ross, G. F. Measurement of the Intrinsic Properties of Materials by Time-Domain Techniques. *IEEE Trans*. 19, 377-382 (1970).

# 2. Matsumoto, M., & Miyata, Y. Thin electromagnetic wave absorber for quasi-microwave band containing aligned thin magnetic metal particles. *IEEE Trans. Magn*. 33, 4459-4464 (1997).

# 3. Wang, X.Y. et al. Mechanism and process of methylene blue degradation by manganese oxides under microwave irradiation. *Appl. Catal. B: Environ.* 160-161, 211-216 (2014).

# 4. Polverejan, M., Villegas, J.C., & Suib, S.L. Higher valency ion substitution into the manganese oxide framework. *J.AM. Chem. Soc*. 126, 7774-7775 (2004).

1. ^1^Beijing Key Laboratory of Materials Utilization of Nonmetallic Minerals and Solid Wastes, National Laboratory of Mineral Materials, School of Materials Science and Technology, China University of Geosciences, Beijing100083, PR China. ^2^Department of Earth Sciences, National Cheng Kung University, Tainan, Taiwan 70101. ^3^Geosciences Department, University of Wisconsin – Parkside, Kenosha, WI 53144, U.S.A. Correspondace and requests for materials should be addressed to G.L. (email: [guochenglv@cugb.edu.cn](mailto:guochenglv@cugb.edu.cn)) or Z.L. (email: li@uwp.edu) [↑](#footnote-ref-2)
